# 

**Published:** 1845-06

**Authors:** 


### CONTENTS

OF THE

### MEDICAL EXAMINER

NEW SERIES—NO. VI.—JUNE, 1845.

---

### ORIGINAL COMMUNICATIONS.

Further Remarks on the Iodide of Potassium in Pneumonia. By George L. Upshur, M. D., of Norfolk, Virginia, - - - - 323

Notes of an Anomalous Case of Traumatic Tetanus, which terminated favourably. By Edward R. Squibb, M.D. - - - - - 328

Winter Climate of St. Mary's. By Alfred Cook, M. D., of Delphi, Onondagua Co., New York. (Communicated by Professor Dunglison,) - - - - - - - - - - - - - 335

### CLINICAL LECTURES AND REPORTS.

PHILADELPHIA HOSPITAL,

*Saturday, January* 25, 1845.

Clinic of Professor Dunglison.

Ramollissement of the Brain and consequent Hemorrhage, - - 338

Hemiplegia, - - - - - - - - - - - - 341

Jerking Respiration, - - - - - - - - - - - - 344

Pathological Specimens, - - - - - - - - - - 345

### BIBLIOGRAPHICAL NOTICES.

Sydenham Society. The seven books of Paulus Ægineta. Translated from the Greek, with a Commentary embracing a complete view of the knowledge possessed by the Greeks, Romans, and Arabians on all subjects connected with Medicine and Surgery. By Francis Adams. In three volumes, - - - - - - - 347

Sydenham Society. Observations on Aneurism, selected from the Works of the Principal writers on the Disease, from the earliest periods to the close of the last century. Translated and edited by John E. Erichsen, Lecturer on General Anatomy and Physiology, at the Westminster Hospital, - - - - - - - 347

A Synopsis of the Symptoms, Diagnosis and Treatment of the more common and important Diseases of the Skin. With sixty coloured Figures. By N. Worcester, M. D., Professor of Physical Diagnosis and General Pathology in the Medical School of Cleveland, late Professor in the Medical College of Ohio, - - 350

Researches on Scrofulous Diseases. By J. G. A. Lugol, D. M. P., Physician to the Hospital St. Louis, Chevalier of the Legion of Honor, &c. &c. Translated from the French. By A. Sidney Doane, A. M., M. D., late Health Officer of the Port of New York, Translator of Meckel's Anatomy, Bayle's Descriptive Anatomy, Blandin's Topographical Anatomy, Dupuytren's Surgery, Maygrier's Midwifery, Scoutetten on Cholera, Ricord on Syphilis, Chaussier on the Arteries, &c., and Editor of Good's Study of Medicine, Surgery Illustrated, &c. &c. &c. With an appendix, comprising formulæ for the treatment of scrofula, - 354

The Philosophy of the moving powers of the Blood. By G. Calvert Holland, M. D., Physician Extraordinary to the Sheffield General Infirmary; formerly President of the Hunterian and Royal Physical Societies, Edinburgh; and Bachelor of Letters of the University of Paris. (With a motto,) - - - - - - 356

A Treatise upon the Diseases and Hygiène of the Organs of the Voice. By Colombat de l'Isere, Chevalier of the Order of the Legion of

Honor, Doctor of Medicine, Founder of the Orthophonic Institute of Paris for the treatment of all vices of speech, Diseases of the Voice, &c. Translated by J. F. W. Lane, M. D. - - 357
Experimental Researches upon Febrile Caloricity, both before and after Death.—Post Mortem Fever. By Bennet Dowler, M. D., of New Orleans. - - - - - - - - - - - 359

### RECORD OF MEDICAL SCIENCE.

Note on Valerianate of Zinc. By William Procter, Jr. - - - 361
On the Action of Lead in distilled and River Water. By Richard Phillips, Junr. - - - - - - . - - - 362
Medical matters in Prussia, - - - - - - - - - - 364
The Diagnosis of Hepatitis and Hepatalgia, - - - - - - 366
On unavoidable Hemorrhage. By Professor Simpson, of the University of Edinburgh, - - - - - - - - - - , 367
Case of Procidentia Uteri during Labour, in which artificial means were necessary to effect Delivery, with subsequent replacement of the Uterus, and complete Recovery. By John M. B. Harden, M. D., of Liberty County, Georgia, - - - - - - - 367
A Case of Lithotomy in the Female—double Calculus. By B. W. Groce, M. D., of Talladega County, Alabama, - - - - 369
Royal Medical and Chirurgical Society, April 8.
Case of obstruction of the Large Intestine, in which the Ascending Colon was opened with success, the patient dying three months afterwards of another Disease. By Samuel Evans, Esq., of Derby, - - - - - - - - - - - - - 371
Aneurismal Tumour of Bone, - - - - - - - - - 375
Academie des Sciences.—February,
The Epizootic in Germany, - - - - - - - - - 377
On the Re-establishment of Nervous Action after Autoplastic Operations, - - - - - - - - - - - 377
Toxical Effects of the Sulphate of Quinine - - - - - - 377

Simple Method of Arresting Hemorrhage from Leech-Bites, - - 378
Surgical Society of Ireland, March 15, 1845.
Case of Intus-susception of the Small Intestine, - - - - - 379
Toxical Effects of Copper and Zinc, - - - - - - - - 378
Effects of Compressed Air, - - - - - - - - - - 378
Experimental Researches on the Action of Oils on the Animal Economy. By MM. Gluge and Thiernesse, - - - - - - 380
Experiments on the Mass of Blood relative to the Weight of the Body. By M. Wanner, - - - - - - - - - - - 381
On the Modification which the Blood undergoes during Inflammation. By MM. Robert-Latour and Collignon, - - - - - - 382
Mortality attending the Operation of Tying the Large Artaries. By T. Inman, of Liverpool, - - - - - - - - - - 383
On the Nature of the Corpora Lutea, and on the difference which they present according as the expulsion of the ovule has been followed or not by conception. By M. Raciborski, - - - - - 384
Sojourn of a Cherry stone nine months in the Air Passages. By M. Maslieurat-Lagemard, - - - - - - - - - - 385
Peculiar form of Dissecting Aneurism of the Thoracic Aorta, - - 385
Possibility of Inflammation in Cold-Blooded Animals, - - - - 386
Febrile Periodicity, as influenced by the Sulphate of Quinine; by Thos. D. Mitchell, M. D., Prof. Mat. Med. and Therap., in Transylvania University, - - - - - - - - - - - - 386
The Seventy-second Anniversary of the Medical Society of London, - 387
The "Cap and Gown," - - - - - - - - - - - - 387
Death of M. Ribes, - - - - - - - - - - - - - 387
Death of M. Ollivier (D'Angers, - - - - - - - - - 388

